# Persistent High Percentage of HLA-DR^+^CD38^high^ CD8^+^ T Cells Associated With Immune Disorder and Disease Severity of COVID-19

**DOI:** 10.3389/fimmu.2021.735125

**Published:** 2021-09-09

**Authors:** Juan Du, Lirong Wei, Guoli Li, Mingxi Hua, Yao Sun, Di Wang, Kai Han, Yonghong Yan, Chuan Song, Rui Song, Henghui Zhang, Junyan Han, Jingyuan Liu, Yaxian Kong

**Affiliations:** ^1^Beijing Key Laboratory of Emerging Infectious Diseases, Institute of Infectious Diseases, Beijing Ditan Hospital, Capital Medical University, Beijing, China; ^2^Beijing Ditan Hospital, Capital Medical University, Beijing, China; ^3^Intensive Care Medicine, Beijing Ditan Hospital, Capital Medical University, Beijing, China; ^4^Clinical and Research Center of Infectious Diseases, Beijing Ditan Hospital, Capital Medical University, Beijing, China

**Keywords:** COVID-19, HLA-DR, CD38, severity, immune disorder

## Abstract

**Background:**

The global outbreak of coronavirus disease 2019 (COVID-19) has turned into a worldwide public health crisis and caused more than 100,000,000 severe cases. Progressive lymphopenia, especially in T cells, was a prominent clinical feature of severe COVID-19. Activated HLA-DR^+^CD38^+^ CD8^+^ T cells were enriched over a prolonged period from the lymphopenia patients who died from Ebola and influenza infection and in severe patients infected with SARS-CoV-2. However, the CD38^+^HLA-DR^+^ CD8^+^ T population was reported to play contradictory roles in SARS-CoV-2 infection.

**Methods:**

A total of 42 COVID-19 patients, including 32 mild or moderate and 10 severe or critical cases, who received care at Beijing Ditan Hospital were recruited into this retrospective study. Blood samples were first collected within 3 days of the hospital admission and once every 3–7 days during hospitalization. The longitudinal flow cytometric data were examined during hospitalization. Moreover, we evaluated serum levels of 45 cytokines/chemokines/growth factors and 14 soluble checkpoints using Luminex multiplex assay longitudinally.

**Results:**

We revealed that the HLA-DR^+^CD38^+^ CD8^+^ T population was heterogeneous, and could be divided into two subsets with distinct characteristics: HLA-DR^+^CD38^dim^ and HLA-DR^+^CD38^hi^. We observed a persistent accumulation of HLA-DR^+^CD38hi CD8^+^ T cells in severe COVID-19 patients. These HLA-DR^+^CD38^hi^ CD8^+^ T cells were in a state of overactivation and consequent dysregulation manifested by expression of multiple inhibitory and stimulatory checkpoints, higher apoptotic sensitivity, impaired killing potential, and more exhausted transcriptional regulation compared to HLA-DR^+^CD38^dim^ CD8^+^ T cells. Moreover, the clinical and laboratory data supported that only HLA-DR^+^CD38^hi^ CD8^+^ T cells were associated with systemic inflammation, tissue injury, and immune disorders of severe COVID-19 patients.

**Conclusions:**

Our findings indicated that HLA-DR^+^CD38^hi^ CD8^+^ T cells were correlated with disease severity of COVID-19 rather than HLA-DR^+^CD38^dim^ population.

## Introduction

Coronavirus disease 2019 (COVID-19) started as an epidemic in Wuhan in 2019 and has become a pandemic (1–3). It rapidly triggered a worldwide public health crisis. As of June, 28, 2021, a total of 180,654,652 cases were identified to be infected by severe acute respiratory syndrome coronavirus 2 (SARS-CoV-2), with 3,920,463 fatal cases, according to the data from WHO. Although the earliest vaccines are already being rolled out in a host of countries, herd immunity to COVID-19 might be very difficult to achieve with current vaccines (4–7). In this case, the spread of SARS-CoV-2 infection would be kept out of control.

Consistent with other respiratory viral infections, adaptive immune responses, particularly cytotoxic T cells, play a vital role in SARS- CoV-2 infection (8–10). It remains a puzzle whether T cell responses in COVID-19 patients are moderate, excessive, or dysfunctional, with evidences provided for all ends of the spectrum. Numerous studies indicated that progressive lymphopenia, especially in T cells, might be highly involved in the pathological process of SARS-CoV-2 infection (11–14). Co-expression of Human Leukocyte Antigen DR (HLA-DR) and CD38 associated with activation of CD8^+^ T cells was reported to accumulate over a prolonged period from the lymphopenia patients who died from Ebola and influenza infection (15–19). These activated HLA-DR^+^CD38^+^ CD8^+^T cells were also noted in mild/moderate and severe cases of COVID-19 patients and displayed a tight correlation with severity of COVID-19 (20–22). However, several studies indicated that CD38^+^HLA-DR^+^CD8^+^ T cells could play a recovery role of activating immunity and eliminating the virus (23, 24). It seemed that the effects of CD38^+^HLA-DR^+^ CD8^+^ T cells in COVID-19 patients varied widely in different studies. Nevertheless, little is known about phenotype and function of CD38^+^HLA-DR^+^ CD8^+^ T cells and association with clinical outcome in COVID-19 patients.

Here, we revealed that the HLA-DR^+^CD38^+^ CD8^+^ T population is heterogeneous of two subpopulations, HLA-DR^+^CD38^dim^ and HLA-DR^+^CD38^hi^ with distinct characteristics.

Furthermore, we found that elevated fraction of HLA-DR^+^CD38^hi^ rather than HLA-DR^+^CD38^+^ CD8^+^ T cells were persistently accumulated in COVID-19 patients, especially in severe and critical cases. These HLA-DR^+^CD38^hi^ CD8^+^ T cells existed in an overactivated and consequently immune disordered state, with high expression of several coinhibitory and costimulatory molecules. This population displayed increased apoptotic sensitivity, impaired killing potential, and more exhausted phenotype and transcriptional regulation, compared to HLA-DR^+^CD38^dim^ CD8^+^ T cells. Of note, the clinical and laboratory data support the notion that HLA-DR^+^CD38^hi^ CD8^+^ T cells were correlated with disease severity of COVID-19 rather than HLA-DR^+^CD38^dim^ population.

## Materials and Methods

### Patients

A total of 42 COVID-19 patients in this retrospective cohort study were enrolled from Beijing Ditan Hospital from March 13, 2020 to April 25, 2020. All enrolled patients were confirmed to be infected with SARS-CoV-2 by RT-PCR assays. This study was approved by the Committee of Ethics at Beijing Ditan Hospital, Capital Medical University. M/M patients were mild and moderate patients, and S/C patients were severe and critical patients, according to the guidelines on the diagnosis and treatment of new coronavirus pneumonia (version 7) by the National Health Commission of China issued on March 3, 2020. These 42 patients included 32 mild or moderate (M/M) patients and 10 severe or critical (S/C) patients. All baseline medical record information including demographic data and clinical characteristics were obtained within the first day after hospital admission (**Table S1**). Blood samples were first collected within 3 days of the hospital admission and once every 3–7 days during hospitalization. The median age of the patients was 37 years (range 20–75) with 50% men and 50% women. Among these 42 patients, the most common were hypertension (five cases), diabetes (three cases), chronic pulmonary disease (three cases), chronic kidney disease (one case), cardiovascular disease (one case). Other clinical details are shown in **Table S1**.

### Ethics

This study was approved by Committee of Ethics at Beijing Ditan Hospital, Capital Medical University [NO. JDLKZ (2020) D (036)-01] with informed consents acquired from all enrolled patients. This study complied with all relevant ethical regulations for work with human participants, and informed consent was obtained. Samples were collected from patients who provided informed consent to participate in the study.

### Peripheral Blood Mononuclear Cells and Serum Isolation

The PBMCs were collected in EDTA at the indicated time points. PBMCs were separated by density gradient centrifugation with lymphocyte separation solution. Serum samples were collected in serum separation tube. The blood was centrifuged at 2,000 rpm for 10 min at 20°C, and the serum was stored at −80°C and thawed at the time of assays. All samples were processed and analyzed within 24 h of collection.

### Flow Cytometric Analysis

PBMCs were incubated with directly conjugated antibodies for 30 min at 4°C. The cells were then washed before flow cytometric analysis. Antibodies used were anti-human CD3-BUV737, CD4-BUV395, PD-1-BV711, CD38-FITC, GITR-BV605 (BD Biosciences, San Diego, CA, USA), CD8-BV510, CTLA-4-BV786, OX40-APC-Fire750, 4-1BB-BV421, HLA-DR-AF700 (BioLegend, San Diego, CA, USA), TIGIT- PE-Cy7, LAG-3-APC, ICOS-PE, (Ebioscience, San Diego, CA, USA), and the corresponding isotype controls. Data acquisition was performed on an LSR Fortessa flow cytometer (BD Biosciences), and data analysis was performed using FlowJo Software (Tree Star, Ashland, OR, USA).

### Intracellular Staining

PBMCs, isolated as described above, were resuspended to 1×10^6^ cells/ml in PBS. The cells were surface-stained with CD3-BV786, CD38-BUV737, HLA-DR-PE, CCR7-BV421, CD45RA-AF700, CD71-APC-H7 (BD), CD4-APC-Fire750, CD8-BV510 (BioLegend) for 30 min in the dark at 4°C, followed by fixation and permeabilization. After permeabilization, cells were stained with ki67-FITC, Granzyme B-AF700, T-bet-BV421, BAX-FITC, Bcl2-PE (BD Biosciences), Eomes-PE-Cy7 (Ebioscience), perforin-APC (BioLegend) antibodies for 30 min in the dark at room temperature. Following staining, cells were washed and acquired on an LSRFortessa.

### 45 Cytokines/Chemokines/Growth Factors and 14 Soluble Checkpoints Multiplex Assay

The serum of 27 COVID-19 patients were assayed for the two multiplexed bead immunoassays. First, we tested 45 ProcartaPlex Human Cytokine/Chemokine/Growth Factor Panel (Invitrogen, Calsbad, CA, USA), including BDNF, Eotaxin/CCL11, EGF, FGF-2, GM-CSF, GROα/CXCL1, HGF, NGFβ, LIF, IFNα, IFNγ, IL-1β, IL-1α, IL-1Rα, IL-2, IL-4, IL-5, IL-6, IL-7, IL-8/CXCL8, IL-9, IL-10, IL-12p70, IL-13, IL-15, IL-17α, IL-18, IL-21, IL-22, IL-23, IL-27, IL-31, IP-10/CXCL10, MCP-1/CCL2, MIP-1α/CCL3, MIP1β/CCL4, RANTES/CCL5, SDF-1α/CXCL12, TNFα, TNFβ/LTA, PDGF-BB, PLGF, SCF, VEGF-A, and VEGF-D. Second, we tested the 14 ProcartaPlex Human ImmunoOncology Checkpoint Panel (Invitrogen), including BTLA, GITR, HVEM, IDO, LAG-3, PD-1, PD-L1, PD-L2, TIM-3, CD28, CD80, 4-1BB, CD27, and CD152. All data were acquired according to the manufacturer’s protocol using Luminex MAGPIX^®^ instrument (Luminex Co., Austin, TX, USA) and analyzed using ProcartaPlex Analyst 1.0 software (Invitrogen).

### Statistical Analysis

GraphPad5 (GraphPad Software, La Jolla, CA, USA) or SPSS (IBM Corporation, New York, NY, USA) was used for statistical calculations. Data are expressed as the mean ± standard deviation (SD) and percentage (frequency), and the normality of each variable was evaluated using the Kolmogorov–Smirnov test. In cases of two normally distributed data, the comparison of variables was respectively performed using unpaired or paired two-tailed Student’s t tests. One-way ANOVA test followed by Tukey’s multiple comparisons test or Holm-Sidak’s multiple comparisons test was performed for comparing two more independent or matched samples. When the data were not normally distributed, the comparison of variables was performed with a Mann–Whitney U test or a Wilcoxon matched-pairs signed-rank test for unpaired and paired data, respectively. For comparing two more samples, a Kruskal–Wallis test or Friedman test followed by Dunn’s multiple comparisons test was applied for independent and matched samples. Comparisons of patient characteristics were analyzed using Fisher’s exact test (categorical variables) or Kruskal–Wallis test (continuous variables). P and correlation coefficient values were obtained using the Spearman’s correlation test. For all analyses, P values <0.05 were considered statistically significant.

## Results

### COVID−19 Cohort

We recruited 42 confirmed COVID-19 patients who received care at Beijing Ditan Hospital. The clinical courses of these cases included 32 mild or moderate (M/M) and 10 severe or critical (S/C) cases have been described in **Table S1**. Twenty age- and gender-matched healthy donors (HDs) were enrolled as controls. Blood samples were first collected within 3 days of the hospital admission and once every 3–7 days during hospitalization. The study was approved by the Committee of Ethics at Beijing Ditan Hospital, Capital Medical University, Beijing, China.

### Persistent Elevated HLA-DR^+^CD38^hi^ CD8^+^ T Cells in S/C Group COVID-19 Cases

**Figure 1:**
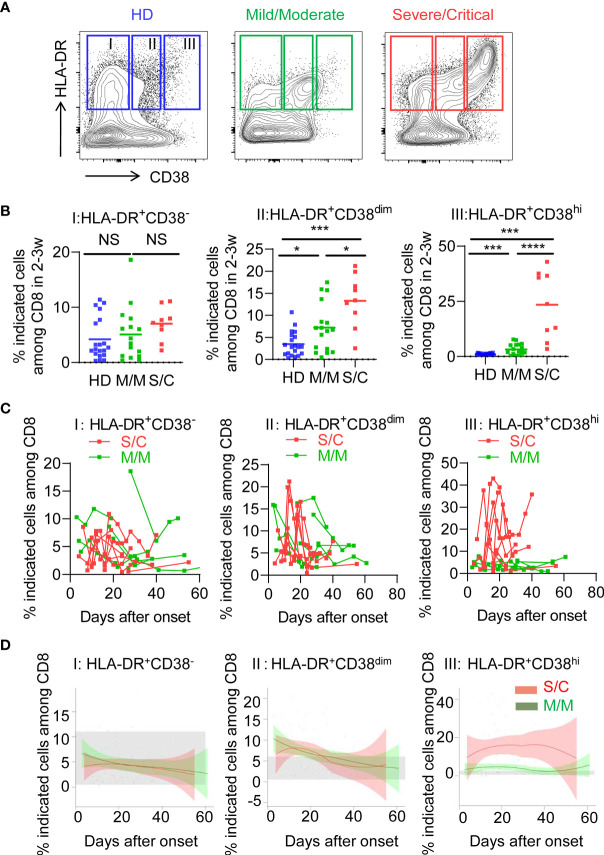
Elevated HLA-DR^+^CD38^hi^ CD8^+^ T cells during acute infection of COVID-19. Flow cytometry analysis of HLA-DR and CD38 expression was performed on PBMCs collected from healthy donors, M/M and S/C patients with COVID-19 infection. **(A)** Representative FACS contour plots showed three subpopulations of HLA-DR^+^ CD8^+^ T cells from healthy donor and COVID-19 patients: HLA-DR^+^CD38^−^ (I), HLA-DR^+^CD38^dim^ (II), HLA-DR^+^CD38^hi^ (III). **(B)** Scatter dot plots of the three percentages of HLA-DR^+^ CD8^+^ T cells from healthy donors and COVID-19 patients within 2–3 weeks post onset (n = 9–20 each group). P Values were obtained by unpaired two-tailed Student’s t tests and Mann–Whitney U test and repeated measures by one-way ANOVA or Kruskal-Wallis test followed by Tukey’s or Dunn’s multiple comparisons test. *P < .05, **P < .01, ***P < .001, ****P < .0001. **(C)** Longitudinal data of three subpopulations were graphed for eight S/C and seven M/M patients with three time points at least. **(D)** Temporal changes of three subpopulations in M/M (n =32) and S/C (n = 10) groups during hospitalization were shown. The 95% confidence interval indicated by colored areas. The normal range of each population was gray shaded region.

To determine the activated status of CD8^+^ T cells, we first analyzed co-expression of HLA-DR and CD38, which are key markers of CD8^+^ T cell activation during viral infection. Based on expression of CD38, three subpopulations were defined among activated HLA-DR^+^CD8^+^ T cells: HLA-DR^+^CD38^−^ (fraction I), HLA-DR^+^CD38^dim^ (fraction II), HLA-DR^+^CD38^hi^ (fraction III), as shown in **Figure 1A**. Patients of S/C group were found to have an obvious peak in HLA-DR^+^CD38^hi^ CD8^+^ T cell (fraction III) within 2–3 weeks post onset (**Figures S1A, B**). We then observed a significantly higher percentage of HLA-DR^+^CD38^hi^ CD8^+^ T cells in all patients including M/M and S/C groups at the peak point, compared to healthy controls. Moreover, the percentage of HLA-DR^+^CD38^hi^ CD8^+^ T cells was dramatically higher in S/C patients than M/M patients (23.48 *vs.* 3.203%, **Figure 1B**). HLA-DR^+^CD38^dim^ CD8^+^ T cells showed a similar trend but less increase. Consequently, the ratio of HLA-DR^+^CD38^dim^ to HLA-DR^+^CD38^hi^ CD8^+^ T cells was significantly higher in M/M than S/C COVID-19 patients in CD8 T cells (**Figure S2**). In contrast, no significant difference was found in HLA-DR^+^CD38^−^ subset among three groups.

We further examined the longitudinal flow cytometric data in eight S/C and eight M/M cases. S/C patients developed elevated HLA-DR^+^CD38^hi^ CD8^+^ T cells early in the infection and displayed persistently high percentage of this population (peak 43%) during the whole course of hospitalization. In contrast, percentage of HLA-DR^+^CD38^hi^ CD8^+^ T cell in M/M cases increased slightly and transiently in the course of illness. As expected, there were no differences in kinetics of HLA-DR^+^CD38^−^ and HLA-DR^+^CD38^dim^ CD8^+^ T cells between S/C and M/M patients (**Figure 1C**). Furthermore, we combined all flow data of each patient (32 M/M and 10 S/C cases) and plotted their fluctuation patterns against the time point post onset. Consistently, these aggregating data showed that percentage of HLA-DR^+^CD38^hi^ CD8^+^ T cells rather than the other two subsets was persistently higher in S/C patients than in M/M cases during hospitalization (**Figure 1D**). Meanwhile, we observed no significant changes of HLA-DR^+^CD38^hi^ CD4^+^ T cells in S/C and M/M patients with COVID-19 infection (**Figure S3**). Overall, our data showed that persistent accumulation of HLA-DR^+^CD38^hi^ CD8^+^ T cells was associated with severity of COVID-19.

### Elevation of HLA-DR^+^CD38^hi^ CD8^+^ T Cells Correlated With Immune Disorders and Tissue Injury in COVID-19 Patients

Next, we applied the longitudinal data of all patients and analyzed the correlation between dynamic changes of circulating HLA-DR^+^CD38^hi^ CD8^+^ T cells and laboratory parameters (**Table 1**). We found percentage of HLA-DR^+^CD38^hi^ CD8^+^ T cells was negatively correlated with absolute counts of lymphocytes, total T cells, CD4, CD8 T cells, B cells, and NK cells, but not neutrophil and monocyte counts. We also observed significant negative correlations between percentage of HLA-DR^+^CD38^hi^ CD8^+^ T cells and hemoglobin (R=−0.546, P<0.0001). Additionally, coagulation-related parameters including platelet count, D-dimer, prothrombin time (PT), and activated partial thromboplastin time (APTT) were detected. We showed that percentage of HLA-DR^+^CD38^hi^ CD8^+^ T cells was significantly associated with D-dimer, which was indicated to correlate with COVID-19 severity (R=0.452, P=0.0003).

**Table 1:** Correlations between CD38^hi^HLA-DR^+^ percentage and parameters in COVID-19 patients.

Characteristics	R value	P values
Immunological parameters		
WBC (×10^9^/L)	0.256	0.0065
Lymphocyte (×109/L)	−0.515	0.0005
T cell (cells/ul)	−0.458	0.0023
CD4 T cell (cells/ul)	−0.394	0.0097
CD8 T cell (cells/ul)	−0.427	0.0047
B cell (cells/ul)	−0.380	0.0155
NK cell (cells/ul)	−0.440	0.0045
Neutrophil (×109/L)	0.326	0.0004
Monocyte (×109/L)	0.030	0.7567
Hemoglobin (g/L)	−0.546	<0.0001
Hematocrit%	−0.552	<0.0001
Other parameters		
Platelets (×109/L)	0.130	0.1714
D-dimer (mg/L)	0.452	0.0003
PT (s)	0.288	0.0189
APTT(s)	0.126	0.333
CRP (mg/L)	0.475	<0.0001
SAA (mg/L)	0.565	<0.0001
AST (U/L)	0.397	<0.0001
Total bilirubin (mmol/L)	0.398	<0.0001
Albumin (g/L)	−0.481	<0.0001
ALT (U/L)	0.059	0.5661
LDH (U/L)	0.643	<0.0001
Serum creatinine (μmol/L)	0.354	0.0003
Creatine kinase (U/L)	0.115	0.3215
Blood potassium (mmol/L)	0.256	0.0071
Blood sodium (mmol/L)	0.078	0.4175

We further found positive correlations between HLA-DR^+^CD38^hi^ CD8^+^ T cells and levels of C-response protein (CRP) and serum amyloid A (SAA), suggesting systemic inflammation (R= 0.475, P<0.0001; R=0.565, P<0.0001). Moreover, the percentage of HLA-DR^+^CD38^hi^ CD8^+^ T cells was found to have positive correlations with aspartate transaminase (AST) and total bilirubin (TB) in COVID-19 patients (R=0.397, P<0.0001; R=0.398, P<0.0001), and a strong negative correlation with albumin (R=−0.481, P<0.0001). These data suggested that high percentage of HLA-DR^+^CD38^hi^ CD8^+^ T cells was involved in liver injury induced by COVID-19. Consistently, levels of lactate dehydrogenase (LDH) and creatinine (CRE) were correlated with the percentage of HLA-DR^+^CD38^hi^ CD8^+^ T cells, respectively (R=0.643, P<0.0001; R=0.354, P=0.0003), indicating myocardial and renal injury (**Table 1**). HLA-DR^+^CD38^dim^ and HLA-DR^+^CD38^−^ CD8 T cells showed no correlation with the clinical characteristics above. Collectively, these results suggested the involvement of HLA-DR^+^CD38^hi^ CD8^+^ T cells in immune disorders and tissue injury in COVID-19 patients.

### Phenotypic and Functional Characterization of HLA-DR^+^CD38^hi^ CD8^+^ T Cells

**Figure 2:**
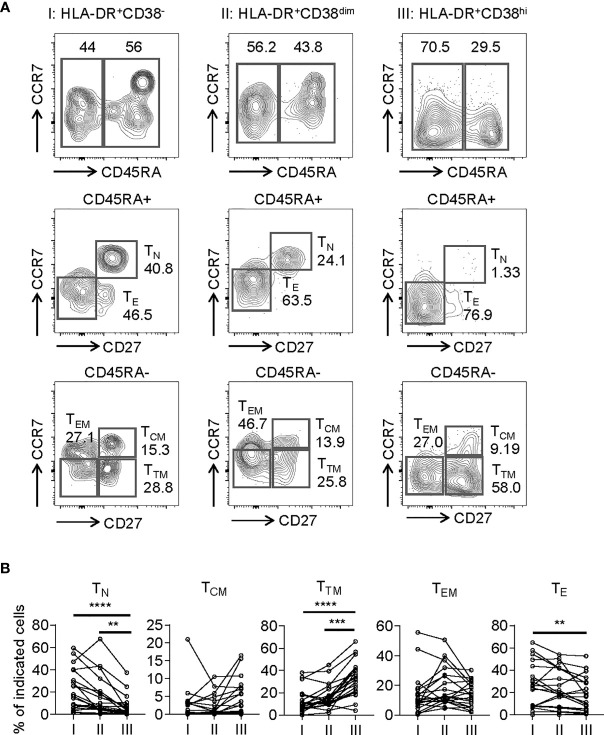
HLA-DR^+^CD38^hi^ CD8^+^ T cells consisted of enhanced percentage of T_TM_ and decreased T_N_ and T_E_. Flow cytometry analysis of T_N_, T_CM_, T_TM_, T_EM_, and T_E_ frequency was performed on PBMCs collected from patients with infection of COVID-19 (n = 20). **(A)** Gating strategy for T_N_, T_CM_, T_TM_, T_EM_, and T_E_ in three CD8^+^ T populations. **(B)** The percentage of T_N_, T_CM_, T_TM_, T_EM_, and T_E_ on each CD8^+^ T population (I, II, III). P Values were obtained by paired two-tailed Student’s t tests and Wilcoxon matched-pairs signed-rank test and repeated measures by one-way ANOVA or Friedman test followed by Holm-Sidak’s multiple comparisons or Dunn’s multiple comparisons test. **P < .01, ***P < .001, ****P < .0001.

To assess the phenotypic status of HLA-DR^+^CD38^hi^ CD8^+^ T cells from COVID-19 patients, we performed additional stains on selected 20 samples from 18 patients. We determined the developmental stage of HLA-DR^+^CD38^hi^ CD8^+^ T cells through dissecting T cells into naïve (T_N_: CD45RA+, CD27+, CCR7+), central memory (T_CM_: CD45RA−, CD27+, CCR7+), transitional memory (T_TM_: CD45RA−, CD27+, CCR7−), effector memory (T_EM_: CD45RA−, CD27−, CCR7−), and effector T cells (T_E_: CD45RA+, CD27−, CCR7−). HLA-DR^+^CD38^hi^ CD8^+^ T cells consisted of enhanced percentage of T_TM_, constant proportion of T_CM_ and T_EM_, and decreased T_N_ and T_E_ (**Figure 2**).

**Figure 3:**
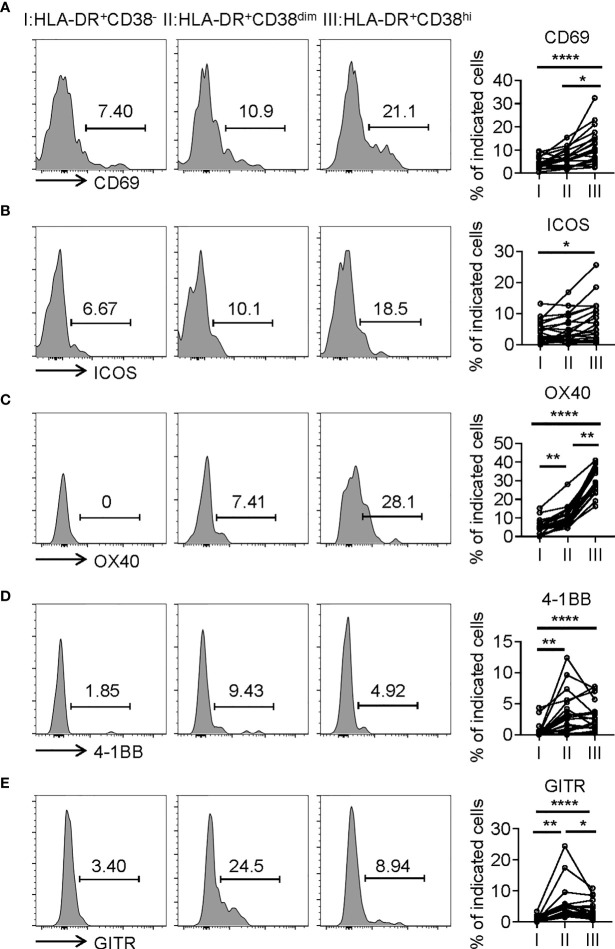
HLA-DR^+^CD38^hi^ CD8^+^ T cells exhibited the phenotype of overactivation. Flow cytometry analysis of expression of CD69 **(A),** ICOS **(B)**, OX40 **(C)**, 4-1BB **(D)**, and GITR **(E)** on three CD8^+^ T populations (I, II, III) from COVID-19 patients (n=20). Representative histograms (left) and plots (right) were shown. P Values were obtained by paired two-tailed Student’s t tests and repeated measures by one-way ANOVA test followed by Holm-Sidak’s multiple comparisons test. *P < .05, **P < .01, ****P < .0001.

**Figure 4:**
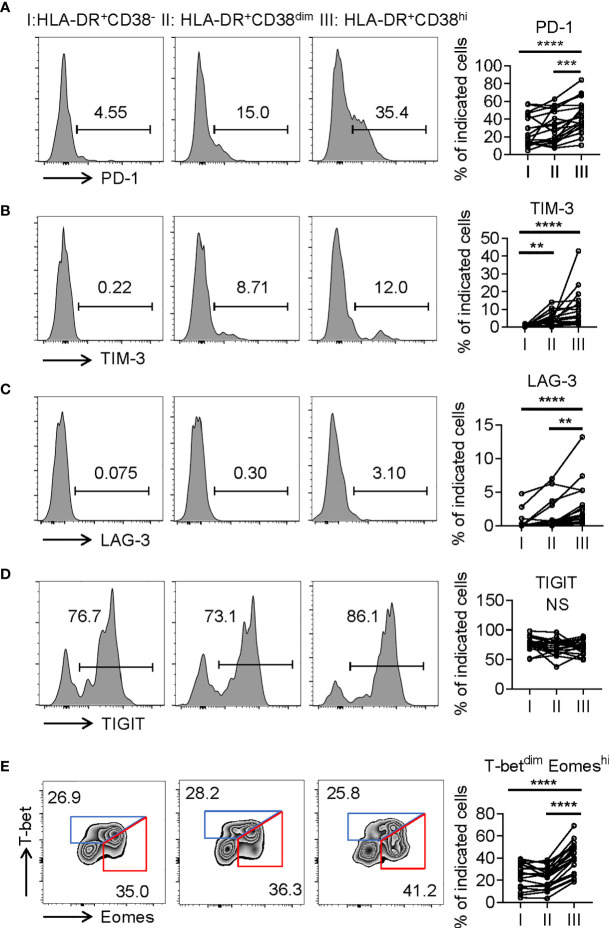
HLA-DR^+^CD38^hi^ CD8^+^ T cells displayed phenotypic and transcriptional state of exhaustion. **(A–D)** Flow cytometry analysis of expression of PD-1 **(A)**, TIM3 **(B)**, LAG3 **(C)**, and TIGIT **(D)** on the three CD8^+^ T population (I, II, III) from COVID-19 patients (n = 20). Representative histograms (left) and plots (right) were shown. **(E)** Representative flow data (left) and dot plots (right) of percentage of T-bet^dim^Eomes^hi^ and T-bet^hi^Eomes^dim^ cells among I, II, III from COVID-19 patients (n = 20). P Values were obtained by paired two-tailed Student’s t tests and repeated measures by one-way ANOVA test followed by Holm-Sidak’s multiple comparisons test. **p < .01, ***p < .001, ****p < .0001.

Consistent with activation, HLA-DR^+^CD38^hi^ CD8^+^ T cells (fraction III) displayed significantly higher levels of CD69, an early activation marker, compared with fractions I and II. We also evaluated expression of costimulatory molecules, including inducible T-cell costimulator (ICOS), OX40, TNF receptor superfamily member 9 (4-1BB), and glucocorticoid-induced tumor necrosis factor receptor (GITR). HLA-DR^+^CD38^hi^ CD8^+^ T cells showed elevated expression of ICOS, OX40, 4-1BB, and GITR compared to fraction I. The level of OX40 in fraction III was higher than in fraction II, while fraction II expressed the highest levels of 4-1BB and GITR (**Figure 3**). Interestingly, we also found HLA-DR^+^CD38^hi^ CD8^+^ T cells expressed higher levels of numerous coinhibitory molecules, including Programmed Death-1 receptor (PD-1), T cell immunoglobulin and mucin-domain containing-3 receptor (TIM-3), LAG-3 (Lymphocyte Activating 3), compared to fractions I and II. No significant difference was found in TIGIT expression of these three fractions. To investigate the intrinsic regulation of HLA-DR^+^CD38^hi^ CD8^+^ T cells, we examined the expression of T-bet and Eomesodermin (Eomes), two key transcription factors governing CD8^+^ T cell exhaustion. We found that HLA-DR^+^CD38^hi^ CD8^+^ T cells contained higher percentage of T-bet^dim^Eomes^hi^ cells, which represented a terminal exhausted status, than fraction I and II. Meanwhile, these three fractions had comparable T-bet^hi^Eomes^dim^ cells (**Figure 4**).

**Figure 5:**
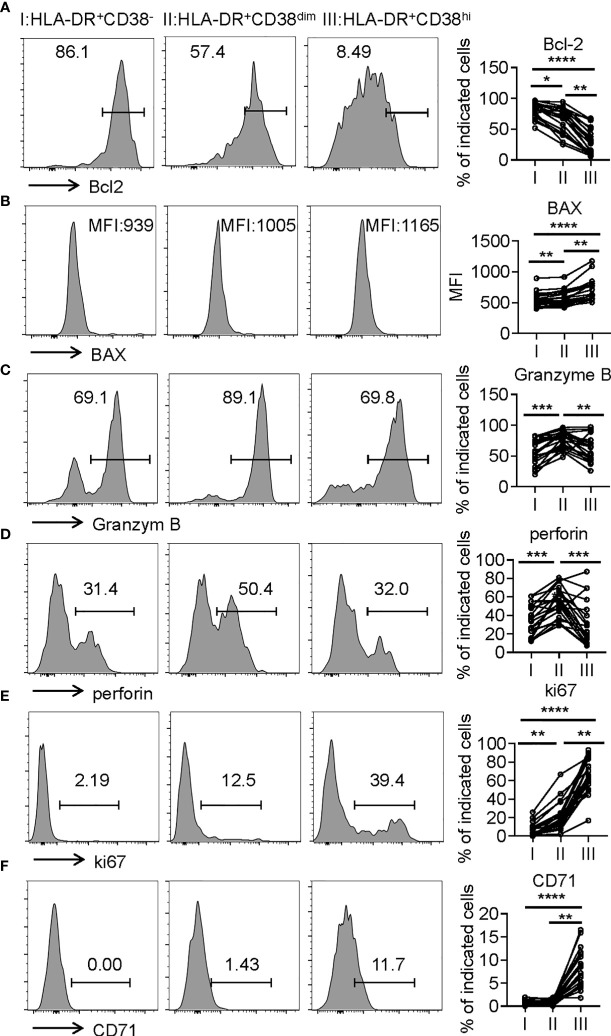
HLA-DR^+^CD38^hi^ CD8^+^ T cells exhibited enhanced susceptibility to apoptosis and highly proliferative potential. Flow cytometry analysis of the expression of Bcl-2 **(A)**, BAX **(B)**, Granzyme B **(C)**, perforin **(D)**, ki67 **(E)**, and CD71 **(F)** on the three CD8^+^ T population (I, II, III) from patients with infection of COVID-19 (n = 20). Representative histograms (left) and plots (right) display the expression of the above receptors on I, II, III. P Values were obtained by paired two-tailed Student’s t tests and repeated measures by one-way ANOVA test followed by Holm-Sidak’s multiple comparisons test. *p < .05, **p < .01, ***p < .001, ****p < .0001.

Consistent with higher frequency of terminally differentiated cells, HLA-DR^+^CD38^hi^ CD8^+^ T cells showed elevated BAX expression and decreased Bcl-2 expression compared with fraction I and II, indicative of high susceptibility to apoptosis. We next investigated killing potential of HLA-DR^+^CD38^hi^ CD8^+^ T cells using intracellular staining of granzyme B and perforin, which are responsible for cytotoxic T lymphocytes to exert their killing function. Distinct from classical exhausted T cells, HLA-DR^+^CD38^hi^ CD8^+^ T cells showed no significant changes of granzyme B and perforin intracellular staining compared with fraction I. Meanwhile, HLA-DR^+^CD38^dim^ CD8^+^ T cells exhibited the highest levels of granzyme B and perforin. Subsequently, we further confirmed that HLA-DR^+^CD38^hi^ CD8^+^ T cells are highly proliferative by expressing higher levels ki67 and CD71 (**Figure 5**). In all, these data suggested an overactivated and consequently disordered immune status of HLA-DR^+^CD38^hi^ CD8^+^ T cells during acute COVID-19 infection.

### HLA-DR^+^CD38^hi^ CD8^+^ T Cells Correlated With Storm of Cytokines and Soluble Checkpoint Molecules

In our previous study, high levels of cytokines and soluble checkpoint molecules were reported to correlate with S/C illness of COVID-19 (25, 26). Due to the disordered immune status of HLA-DR^+^CD38^hi^ CD8^+^ T cells, we wondered the effects of these cells in the storms of cytokines and soluble checkpoint molecules. We evaluated serum levels of 45 cytokines/chemokines/growth factors and 14 soluble checkpoints using Luminex multiplex assay from 27 COVID-19 patients at different time points during hospitalization and collected longitudinal data. Levels of 17 factors such as HGF, IL-18, IL-1RA, MCP-1, RANTES, IL-10, and SCF showed significantly positive correlations with percentage of HLA-DR^+^CD38^hi^ CD8^+^ T cells. Moreover, 10 serum soluble checkpoint molecules, such as TIM3, CD27, IDO, and LAG3, were positively correlated with percentage of HLA-DR^+^CD38^hi^ CD8^+^ T cells. Moreover, two other populations HLA-DR^+^CD38^−^ and HLA-DR^+^CD38^dim^ showed no correlations to storm of cytokines and soluble checkpoint molecules (**Table 2** and **Figure S4**). Thus, we hypothesized that elevated HLA-DR^+^CD38^hi^ CD8^+^ T group might potentially contribute to the storm of cytokine and soluble checkpoint molecules occurring in COVID-19 patients.

**Table 2:** Correlations between CD38^hi^HLA-DR^+^ percentage and soluble immune checkpoints, cytokines, chemokines, and growth factors in patients infected with SARS-CoV2.

Characteristics	R value	P values
Cytokines, chemokines, and growth factors		
HGF	0.576	<0.0001
IL-18	0.530	<0.0001
IL-1RA	0.519	0.00013
MCP-1	0.504	0.00022
RANTES	0.435	0.00179
IL-10	0.403	0.00409
SCF	0.401	0.00429
IL-8	0.392	0.00529
IL-21	0.390	0.00558
IL-1alpha	0.359	0.01128
IFN-gamma	0.342	0.01603
IL-4	0.317	0.02646
SDF-1alpha	0.306	0.03247
IL-22	0.303	0.03431
IL-6	0.299	0.03721
GRO-alpha	0.288	0.04774
BDNF	−0.306	0.03266
Soluble immune checkpoints		
TIM-3	0.549	<0.0001
CD27	0.459	0.00090
LAG-3	0.411	0.00339
IDO	0.401	0.00425
BTLA	0.386	0.00621
CD152	0.361	0.01087
CD137	0.349	0.01411
PD-1	0.346	0.01473
CD80	0.329	0.02095
CD28	0.285	0.04694

## Discussion

Previous studies noted both T cell activation and exhaustion during SARS-CoV-2 infection (10, 14, 27, 28). Although COVID-19 patients did develop severe lymphopenia in response to T cell exhaustion, an elevated proportion of HLA-DR^+^CD38^+^ CD8^+^ T cells suggests a potent adaptive immune response in these patients (29). HLA-DR and CD38 molecules, which are transmembrane glycoproteins, are present on immature T and B lymphocytes and are re-expressed during immune response. Thus, expression of HLA-DR and CD38 respectively on CD8^+^ T cells reflects immune activation. In particular, co-expression of CD38 and HLA-DR on CD8^+^ T cells was regarded as a better marker of immune activation during influenza, Dengue, Ebola, and HIV-1 viral infections (30–35). However, in SARS-CoV-2 infection, HLA-DR^+^CD38^+^ CD8^+^ T cells were reported to play contradictory roles. Severe COVID-19 patients showed a significant increase of HLA-DR^+^CD38^+^ CD8^+^ T cells compared to mild cases (20, 36). In a cohort of critical COVID-19 patients with hypertension, the percentage of CD38^+^HLA-DR^+^ fraction among CD8^+^ T cells was higher in the patients with fatal outcomes compared with the surviving patients (37). These studies suggested the involvement of CD38^+^HLA-DR^+^ CD8^+^ T cells in severe progression of COVID-19. In contrast, about 20% of patients had no increase in CD38^+^HLA-DR^+^ CD8^+^ T cells above the level found in HD (22). A study with a cohort of 6 severe and 11 mild COVID-19 patients found no significant differences of CD38^+^HLA-DR^+^ CD8^+^ T cells between mild and severe patients (36). Furthermore, Wang et al. observed that the number of CD38^+^HLA-DR^+^ CD8^+^ T cells was markedly higher in recovering group than severe persistence group among severe COVID-19 patients (23). Activated CD8^+^ T cells with CD38 signature contributed to the elimination of SARS-CoV-2 in the lungs, indicating a recovery role of these cells for boosting immune response and eliminating virus (24). These contradictory results of CD38^+^HLA-DR^+^ CD8^+^ T cells in COVID-19 patients implied the heterogeneity of this population, which was supported by our findings.

In the present study, we found that HLA-DR^+^CD38^+^ CD8^+^ T cells contained two distinct subpopulations, HLA-DR^+^CD38^hi^ and HLA-DR^+^CD38^dim^. HLA-DR^+^CD38^hi^ CD8^+^ T cells were demonstrated to only accumulate in COVID-19 patients, especially S/C cases. The proportion of HLA-DR^+^CD38^hi^ CD8^+^ T cells was significantly higher in S/C than M/M group. Notably, a high frequency of HLA-DR^+^CD38^hi^ CD8^+^ T cells strongly correlated with severe lymphopenia, systemic inflammation, and tissue injury, suggesting a predictive value of this cell population for disease progression in COVID-19 patients. Conversely, HLA-DR^+^CD38^dim^ CD8^+^ T cells existed in both M/M and S/C patients, even healthy individuals. Additionally, S/C cases had transient elevation of HLA-DR^+^CD38^dim^ CD8^+^ T cells, but a prolonged high percentage of HLA-DR^+^CD38^hi^ fraction. Phenotypic analysis of these two subsets further demonstrated that HLA-DR^+^CD38^+^ CD8^+^ T cells were heterogeneous. It was revealed that HLA-DR^+^CD38^dim^ CD8^+^ T cells expressed low levels of inhibitory checkpoints, high levels of 4-1BB and GITR, stronger killing potential, and weaker sensitivity to apoptosis. Meanwhile, HLA-DR^+^CD38^hi^ CD8^+^ T cells were in a state of overactivation, or exhaustion, manifested by expression of multiple inhibitory and stimulatory checkpoints, more exhausted transcriptional regulation, higher apoptotic sensitivity, and impaired killing potential. Consistently, M/M patients showed a high ratio of HLA-DR^+^CD38^dim^ to HLA-DR^+^CD38^hi^, implying activated immune responses and effective virus clearance, whereas much lower ratio of HLA-DR^+^CD38^dim^ to HLA-DR^+^CD38^hi^ was found in S/C group, representing immune exhaustion, systemic tissue injury, and subsequently poor outcome. Thus, a distinct ratio of these two subsets might contribute to different immune response and clinical outcome of HLA-DR^+^CD38^+^ CD8^+^ T cells in COVID-19 progression.

To our knowledge, HLA-DR^+^CD38^hi^ CD8^+^ T cells were first reported to associate with a series of soluble immune checkpoint molecules, including sTIM3, sCD27, sLAG3, and sIDO. Considering the theory that soluble forms of checkpoint molecules are produced by cleavage of membrane-bound protein or by mRNA expression (38, 39), we supposed that HLA-DR^+^CD38^hi^ CD8^+^ T cell with high expression of membrane-bound molecules contributed to the storm of soluble checkpoint molecules. Our previous study also demonstrated the same soluble molecules as predictive biomarkers for disease severity of COVID-19, further supporting the important role of HLA-DR^+^CD38^hi^ CD8^+^ T cells (25). In addition, elevated levels of soluble checkpoints as well as membrane-bound forms on HLA-DR^+^CD38^hi^ CD8^+^ T cells included stimulatory and inhibitory molecules, which boost potent immune response and maintain self-tolerance. Thus, the total effects of these HLA-DR^+^CD38^hi^ CD8^+^ T cells and heterogeneous checkpoint molecules on immune response are difficultly computable in S/C cases of COVID-19, which reflected a broad and complicated dysregulation of T cell immunity. The severity of COVID-19 might represent a consequence from the imbalance between stimulatory and inhibitory checkpoints.

Both progressive lymphopenia and cytokine release syndrome were prominent clinical features of S/C COVID-19 in addition to dyspnea, hypoxemia, and acute respiratory distress (40–42). As expected, these HLA-DR^+^CD38^hi^ CD8^+^ T cells were positively correlated to numerous inflammatory cytokines, IL-18, IL-10, IL-21, IL-1α, IFN-γ, IL-4, IL-22, and IL-6, implying a dysregulated state of these cells. It was in agreement with the characteristics of these cells. Notably, a few chemokines, including MCP-1, RANTES, and IL-8, showed significantly positive correlations with HLA-DR^+^CD38^hi^ CD8^+^ T cells. Consistently, CD8^+^ T cells were identified in lung and liver tissues from COVID-19 patients by postmortem biopsy in previous studies (24, 43–45). Thus, we speculated that these cells might accumulate in target organs towards chemokines and could be a potential culprit of tissue injury. The notion was supported by a close correlation between the proportion of these cells and clinical parameters of systemic inflammation and tissue injury.

COVID-19 patients including S/C and M/M cases showed elevated percentages of HLA-DR^+^CD38^hi^CD8^+^ T cells up to 60 d after symptom onset. This finding is distinct from the responses of activated CD8^+^ T cells that were found in other acute viral infections, in which the activated T cells returned to baseline much faster (46–48). This implies the persistence of viral antigen continually stimulating these responses. Previous studies demonstrated that the median duration of viral shedding was 20 days in survivors, but SARS-CoV-2 was detectable until death in non-survivors (3). This finding was consistent with the persistently high percentage of HLA-DR^+^CD38^hi^ CD8^+^ T cells from a death in the present study. Surprisingly, despite a respiratory virus, SARS-CoV-2 RNA in rectal samples was found to remain for a long period, with a higher positive rate and higher viral load than the paired respiratory samples. It is worth noting that the longest duration observed was 43 days, much longer than the usual 3–5 weeks from symptom onset to discharge for most patients (49). However, M/M COVID-19 patients showed low but prolonged activated CD8^+^ T cells, which could be explained by the fact that gastrointestinal viral reservoir of SARS-CoV-2 exists persistently even in mild and asymptomatic patients.

Taken together, we found accumulation of a novel HLA-DR^+^CD38^hi^ population instead of heterogeneous HLA-DR^+^CD38^+^ CD8^+^ T cells during SARS-CoV-2 infection, especially in severe and critical cases. These HLA-DR^+^CD38^hi^ CD8^+^ T cells existed in an overactivated and consequently immune disordered state, with high expression of multiple coinhibitory and costimulatory molecules (22, 50). Of note, a high frequency of HLA-DR^+^CD38^hi^ CD8^+^ T cells strongly correlated with severe lymphopenia, systemic inflammation, and storm of cytokines and soluble checkpoint molecules, indicating a predictive value of this cell population for disease progression in COVID-19 patients.

Our study has several limitations, including small sample size, unmatched ages between groups, and variable sampling interval for each patient. More importantly, due to lack of functional data, it is difficult to determine the precise functional characteristics of HLA-DR^+^CD38^hi^ and HLA-DR^+^CD38^dim^ CD8^+^ T cells. Therefore, more evidences are urgently needed to investigate whether these two subsets play distinct roles in the pathogenesis and severity of COVID-19.

## Data Availability Statement

The datasets presented in this study can be found in online repositories. The names of the repository/repositories and accession number(s) can be found in the article/**Supplementary Material**.

## Ethics Statement

The studies involving human participants were reviewed and approved by the Committee of Ethics at Beijing Ditan Hospital. The patients/participants provided their written informed consent to participate in this study.

## Author Contributions

JD performed the experiments, analyzed the data, and wrote the manuscript. LW, GL, and MH collected samples and performed the experiments. DW collected clinical data. KH, YY, and CS collected samples. YS and RS recruited patients. HZ participated in the critical review of the manuscript. JH designed and performed the experiments. JL conducted the study and recruited patients. YK conceived the study, performed the experiments, and wrote the manuscript. All authors contributed to the article and approved the submitted version.

## Funding

This work was supported by the National Natural Science Foundation of China (81971307), Beijing Natural Science Foundation (M21007), Beijing Municipal Science & Technology Commission (Z201100007920017), National Key Sci-Tech Special Project of China (2018ZX10302207), and National Key Research and Development Program of China (2020YFC0846200).

## Conflict of Interest

The authors declare that the research was conducted in the absence of any commercial or financial relationships that could be construed as a potential conflict of interest.

## Publisher’s Note

All claims expressed in this article are solely those of the authors and do not necessarily represent those of their affiliated organizations, or those of the publisher, the editors and the reviewers. Any product that may be evaluated in this article, or claim that may be made by its manufacturer, is not guaranteed or endorsed by the publisher.

## References

[1] Huang C Wang Y Li X Ren L Zhao J Hu Y. Clinical Features of Patients Infected With 2019 Novel Coronavirus in Wuhan, China. Lancet (2020) 395(10223):497–506. 10.1016/S0140-6736(20)30183-5 31986264 PMC7159299

[2] Du Y Tu L Zhu P Mu M Wang R Yang P. Clinical Features of 85 Fatal Cases of COVID-19 From Wuhan. A Retrospective Observational Study. Am J Respir Crit Care Med (2020) 201(11):1372–9. 10.1164/rccm.202003-0543OC PMC7258652 32242738

[3] Zhou F Yu T Du R Fan G Liu Y Liu Z. Clinical Course and Risk Factors for Mortality of Adult Inpatients With COVID-19 in Wuhan, China: A Retrospective Cohort Study. Lancet (2020) 395(10229):1054–62. 10.1016/S0140-6736(20)30566-3 PMC7270627 32171076

[4] Wouters OJ Shadlen KC Salcher-Konrad M Pollard AJ Larson HJ Teerawattananon Y. Challenges in Ensuring Global Access to COVID-19 Vaccines: Production, Affordability, Allocation, and Deployment. Lancet (2021) 397(10278):1023–34. 10.1016/S0140-6736(21)00306-8 PMC7906643 33587887

[5] Dai L Gao GF. Viral Targets for Vaccines Against COVID-19. Nat Rev Immunol (2021) 21(2):73–82. 10.1038/s41577-020-00480-0 33340022 PMC7747004

[6] Forni G Mantovani A. COVID-19 Vaccines: Where We Stand and Challenges Ahead. Cell Death Differ (2021) 28(2):626–39. 10.1038/s41418-020-00720-9 PMC7818063 33479399

[7] Su S Du L Jiang S. Learning From the Past: Development of Safe and Effective COVID-19 Vaccines. Nat Rev Microbiol (2021) 19(3):211–9. 10.1038/s41579-020-00462-y PMC7566580 33067570

[8] Shi Y Wang Y Shao C Huang J Gan J Huang X. COVID-19 Infection: The Perspectives on Immune Responses. Cell Death Differ (2020) 27(5):1451–4. 10.1038/s41418-020-0530-3 PMC7091918 32205856

[9] Chen G Wu D Guo W Cao Y Huang D Wang H. Clinical and Immunological Features of Severe and Moderate Coronavirus Disease 2019. J Clin Invest (2020) 130(5):2620–9. 10.1172/JCI137244 PMC7190990 32217835

[10] Braun J Loyal L Frentsch M Wendisch D Georg P Kurth F. SARS-CoV-2-Reactive T Cells in Healthy Donors and Patients With COVID-19. Nature (2020) 587(7833):270–4. 10.1038/s41586-020-2598-9 32726801

[11] Fathi N Rezaei N. Lymphopenia in COVID-19: Therapeutic Opportunities. Cell Biol Int (2020) 44(9):1792–7. 10.1002/cbin.11403 PMC7283672 32458561

[12] Sekine T Perez-Potti A Rivera-Ballesteros O Strålin K Gorin JB Olsson A. Robust T Cell Immunity in Convalescent Individuals With Asymptomatic or Mild COVID-19. Cell (2020) 183(1):158–68.e14. 10.1016/j.cell.2020.08.017 32979941 PMC7427556

[13] Schultheiß C Paschold L Simnica D Mohme M Willscher E von Wenserski L. Next-Generation Sequencing of T and B Cell Receptor Repertoires From COVID-19 Patients Showed Signatures Associated With Severity of Disease. Immunity (2020) 53(2):442–55.e4. 10.1016/j.immuni.2020.06.024 32668194 PMC7324317

[14] Wilk AJ Rustagi A Zhao NQ Roque J Martínez-Colón GJ McKechnie JL. A Single-Cell Atlas of the Peripheral Immune Response in Patients With Severe COVID-19. Nat Med (2020) 26(7):1070–6. 10.1038/s41591-020-0944-y PMC7382903 32514174

[15] Agrati C Castilletti C Casetti R Sacchi A Falasca L Turchi F. Longitudinal Characterization of Dysfunctional T Cell-Activation During Human Acute Ebola Infection. Cell Death Dis (2016) 7(3):e2164. 10.1038/cddis.2016.55 27031961 PMC4823956

[16] Wang Z Zhu L Nguyen T Wan Y Sant S Quiñones-Parra SM. Clonally Diverse CD38(+)HLA-DR(+)CD8(+) T Cells Persist During Fatal H7N9 Disease. Nat Commun (2018) 9(1):824. 10.1038/s41467-018-03243-7 29483513 PMC5827521

[17] Thom R Tipton T Strecker T Hall Y Akoi Bore J Maes P. Longitudinal Antibody and T Cell Responses in Ebola Virus Disease Survivors and Contacts: An Observational Cohort Study. Lancet Infect Dis (2021) 21(4):507–16. 10.1016/S1473-3099(20)30736-2 PMC7553754 33065039

[18] McElroy AK Akondy RS Davis CW Ellebedy AH Mehta AK Kraft CS. Human Ebola Virus Infection Results in Substantial Immune Activation. Proc Natl Acad Sci U S A (2015) 112(15):4719–24. 10.1073/pnas.1502619112 PMC4403189 25775592

[19] Koutsakos M Illing PT Nguyen T Mifsud NA Crawford JC Rizzetto S. Human CD8(+) T Cell Cross-Reactivity Across Influenza A, B and C Viruses. Nat Immunol (2019) 20(5):613–25. 10.1038/s41590-019-0320-6 30778243

[20] Song JW Zhang C Fan X Meng FP Xu Z Xia P. Immunological and Inflammatory Profiles in Mild and Severe Cases of COVID-19. Nat Commun (2020) 11(1):3410. 10.1038/s41467-020-17240-2 32641700 PMC7343781

[21] Habel JR Nguyen T van de Sandt CE Juno JA Chaurasia P Wragg K. Suboptimal SARS-CoV-2-Specific CD8(+) T Cell Response Associated With the Prominent HLA-A*02:01 Phenotype. Proc Natl Acad Sci U S A (2020) 117(39):24384–91. 10.1073/pnas.2015486117 PMC7533701 32913053

[22] Mathew D Giles JR Baxter AE Oldridge DA Greenplate AR Wu JE. Deep Immune Profiling of COVID-19 Patients Reveals Distinct Immunotypes With Therapeutic Implications. Science (2020) 369(6508). 10.1126/science.abc8511 PMC7402624 32669297

[23] Wang Z Yang X Zhou Y Sun J Liu X Zhang J. COVID-19 Severity Correlates With Weaker T-Cell Immunity, Hypercytokinemia, and Lung Epithelium Injury. Am J Respir Crit Care Med (2020) 202(4):606–10. 10.1164/rccm.202005-1701LE PMC7427397 32608999

[24] Nienhold R Ciani Y Koelzer VH Tzankov A Haslbauer JD Menter T. Two Distinct Immunopathological Profiles in Autopsy Lungs of COVID-19. Nat Commun (2020) 11(1):5086. 10.1038/s41467-020-18854-2 33033248 PMC7546638

[25] Kong Y Wang Y Wu X Han J Li G Hua M. Storm of Soluble Immune Checkpoints Associated With Disease Severity of COVID-19. Signal Transduct Target Ther (2020) 5(1):192. 10.1038/s41392-020-00308-2 32895366 PMC7475713

[26] Kong Y Han J Wu X Zeng H Liu J Zhang H. VEGF-D: A Novel Biomarker for Detection of COVID-19 Progression. Crit Care (2020) 24(1):373. 10.1186/s13054-020-03079-y 32576222 PMC7309201

[27] De Biasi S Meschiari M Gibellini L Bellinazzi C Borella R Fidanza L. Marked T Cell Activation, Senescence, Exhaustion and Skewing Towards TH17 in Patients With COVID-19 Pneumonia. Nat Commun (2020) 11(1):3434. 10.1038/s41467-020-17292-4 32632085 PMC7338513

[28] Paces J Strizova Z Smrz D Cerny J. COVID-19 and the Immune System. Physiol Res (2020) 69(3):379–88. 10.33549/physiolres.934492 PMC8648321 32469225

[29] Kratzer B Trapin D Ettel P Körmöczi U Rottal A Tuppy F. Immunological Imprint of COVID-19 on Human Peripheral Blood Leukocyte Populations. Allergy (2021) 76(3):751–65. 10.1111/all.14647 PMC7984452 33128792

[30] Ruibal P Oestereich L Lüdtke A Becker-Ziaja B Wozniak DM Kerber R. Unique Human Immune Signature of Ebola Virus Disease in Guinea. Nature (2016) 533(7601):100–4. 10.1038/nature17949 PMC4876960 27147028

[31] Radziewicz H Ibegbu CC Hon H Osborn MK Obideen K Wehbi M. Impaired Hepatitis C Virus (HCV)-Specific Effector CD8+ T Cells Undergo Massive Apoptosis in the Peripheral Blood During Acute HCV Infection and in the Liver During the Chronic Phase of Infection. J Virol (2008) 82(20):9808–22. 10.1128/JVI.01075-08 PMC2566282 18667503

[32] Hua S Lécuroux C Sáez-Cirión A Pancino G Girault I Versmisse P. Potential Role for HIV-Specific CD38-/HLA-DR+ CD8+ T Cells in Viral Suppression and Cytotoxicity in HIV Controllers. PloS One (2014) 9(7):e101920. 10.1371/journal.pone.0101920 25000587 PMC4084978

[33] Murray SM Down CM Boulware DR Stauffer WM Cavert WP Schacker TW. Reduction of Immune Activation With Chloroquine Therapy During Chronic HIV Infection. J Virol (2010) 84(22):12082–6. 10.1128/JVI.01466-10 PMC2977889 20844049

[34] Chandele A Sewatanon J Gunisetty S Singla M Onlamoon N Akondy RS. Characterization of Human CD8 T Cell Responses in Dengue Virus-Infected Patients From India. J Virol (2016) 90(24):11259–78. 10.1128/JVI.01424-16 PMC5126381 27707928

[35] Fox A Le NM Horby P van Doorn HR Nguyen VT Nguyen HH. Severe Pandemic H1N1 2009 Infection Is Associated With Transient NK and T Deficiency and Aberrant CD8 Responses. PloS One (2012) 7(2):e31535. 10.1371/journal.pone.0031535 22363665 PMC3282732

[36] Zhou R To KK Wong YC Liu L Zhou B Li X. Acute SARS-CoV-2 Infection Impairs Dendritic Cell and T Cell Responses. Immunity (2020) 53:864–877.e5. 10.1016/j.immuni.2020.07.026 32791036 PMC7402670

[37] Zeng Q Li YZ Dong SY Chen ZT Gao XY Zhang H. Dynamic SARS-CoV-2-Specific Immunity in Critically Ill Patients With Hypertension. Front Immunol (2020) 11:596684. 10.3389/fimmu.2020.596684 33362779 PMC7758245

[38] Gu D Ao X Yang Y Chen Z Xu X. Soluble Immune Checkpoints in Cancer: Production, Function and Biological Significance. J Immunother Cancer (2018) 6:132. 10.1186/s40425-018-0449-0 30482248 PMC6260693

[39] Jalali S Price-Troska T Paludo J Villasboas J Kim HJ Yang ZZ. Soluble PD-1 Ligands Regulate T-Cell Function in Waldenstrom Macroglobulinemia. Blood Adv (2018) 2:1985–97. 10.1182/bloodadvances.2018021113 PMC6093740 30104397

[40] Moore JB June CH. Cytokine Release Syndrome in Severe COVID-19. Science (2020) 368:473–4. 10.1126/science.abb8925 32303591

[41] Hu B Huang S Yin L. The Cytokine Storm and COVID-19. J Med Virol (2021) 93:250–6. 10.1002/jmv.26232 PMC7361342 32592501

[42] Picchianti Diamanti A Rosado MM Pioli C Sesti G Laganà B. Cytokine Release Syndrome in COVID-19 Patients, A New Scenario for an Old Concern: The Fragile Balance Between Infections and Autoimmunity. Int J Mol Sci (2020) 21. 10.3390/ijms21093330 PMC7247555 32397174

[43] Bidari Zerehpoosh F Sabeti S Bahrami-Motlagh H Mokhtari M Naghibi Irvani SS Torabinavid P. Post-Mortem Histopathologic Findings of Vital Organs in Critically Ill Patients With COVID-19. Arch Iran Med (2021) 24:144–51. 10.34172/aim.2021.23 33636984

[44] Ribeiro Dos Santos Miggiolaro,AF da Silva Motta Junior J Busatta Vaz de Paula C Nagashima S Alessandra Scaranello Malaquias M Baena Carstens L. Covid-19 Cytokine Storm in Pulmonary Tissue: Anatomopathological and Immunohistochemical Findings. Respir Med Case Rep (2020) 31:101292. 10.1016/j.rmcr.2020.101292 33200067 PMC7658564

[45] Gauchotte G Venard V Segondy M Cadoz C Esposito-Fava A Barraud D. SARS-Cov-2 Fulminant Myocarditis: An Autopsy and Histopathological Case Study. Int J Legal Med (2021) 135:577–81. 10.1007/s00414-020-02500-z PMC7779100 33392658

[46] Bournazos S Corti D Virgin HW Ravetch JV. Fc-Optimized Antibodies Elicit CD8 Immunity to Viral Respiratory Infection. Nature (2020) 588:485–90. 10.1038/s41586-020-2838-z PMC7672690 33032297

[47] Sridhar S Begom S Bermingham A Hoschler K Adamson W Carman W. Cellular Immune Correlates of Protection Against Symptomatic Pandemic Influenza. Nat Med (2013) 19:1305–12. 10.1038/nm.3350 24056771

[48] Sakabe S Sullivan BM Hartnett JN Robles-Sikisaka R Gangavarapu K Cubitt B. Analysis of CD8(+) T Cell Response During the 2013-2016 Ebola Epidemic in West Africa. Proc Natl Acad Sci U S A (2018) 115:E7578–7578E7586. 10.1073/pnas.1806200115 30038008 PMC6094108

[49] Zhao F Yang Y Wang Z Li L Liu L Liu Y. The Time Sequences of Respiratory and Rectal Viral Shedding in Patients With Coronavirus Disease 2019. Gastroenterology (2020) 159:1158–1160.e2. 10.1053/j.gastro.2020.05.035 32425226 PMC7229722

[50] Rupp J Dreo B Gütl K Fessler J Moser A Haditsch B. T Cell Phenotyping in Individuals Hospitalized With COVID-19. J Immunol (2021) 206:1478–82. 10.4049/jimmunol.2001034 33558375

