# Supplementary material for: Persistent High Percentage of HLA-DR+CD38high CD8+ T Cells Associated With Immune Disorder and Disease Severity of COVID-19
Source: Front Immunol. 2021 Sep 9;12:735125. doi: 10.3389/fimmu.2021.735125 (PMC8458852; doi:10.3389/fimmu.2021.735125)
Supplement: Supplementary file 1 [file DataSheet_1.docx]

Supplementary Materials for

**Persistently High Percentage of HLA-DR^+^CD38^high^CD8^+^T Cells Associated with** **Immune Disorder and Disease Severity of** **COVID-19**

Juan Du^1#^, Lirong Wei^2#^, Guoli Li^1#^, Mingxi Hua^1#^, Yao Sun^3^, Di Wang^4^, Kai Han^1^, Yonghong Yan^1^, Chuan Song^1^, Rui Song^2^, Henghui Zhang^1^, Junyan Han^1^*, Jingyuan Liu^3^*, Yaxian Kong^1^*

*^1^Beijing Key Laboratory of Emerging Infectious Diseases, Institute of Infectious Diseases, Beijing Ditan Hospital, Capital Medical University, Beijing 100015, China*

*^2^Beijing Ditan Hospital, Capital Medical University, Beijing 100015, China*

*^3^Intensive Care Medicine, Beijing Ditan Hospital, Capital Medical University,*

*Beijing 100015, China*

*^4^Clinical and Research Center of Infectious Diseases, Beijing Ditan Hospital, Capital Medical University, Beijing 100015, China*

**^#^**These four authors contributed equally to this work

*Yaxian Kong, Jingyuan Liu, and Junyan Han are co-senior authors and contributed equally to this work.

**Email:** kongyaxian@ccmu.edu.cn, dtyyicu@ccmu.edu.cn, hanjunyan@ccmu.edu.cn

**This Supplementary file includes:**

Figures. S1 to S5 and Tables S1

Figure S1. Representative FACS plots of HLA-DR and CD38 expression were shown at different days post onset during acute infection of COVID-19.

Figure S2. The ratio of HLA-DR^+^CD38^dim^ to HLA-DR^+^CD38^hi^ in CD8 was calculated at 2-3 weeks post onset.

Figure S3. HLA-DR^+^CD38^hi^CD4^+^T cells during acute infection of COVID-19.

Figure S4. Correlation heatmap of three CD8 subsets with 45 cytokines concentrations (left) and soluble checkpoint molecules concentrations (right) were shown.

Tables S1. Demographics and baseline characteristics of patients infected with SARS-CoV2.

**
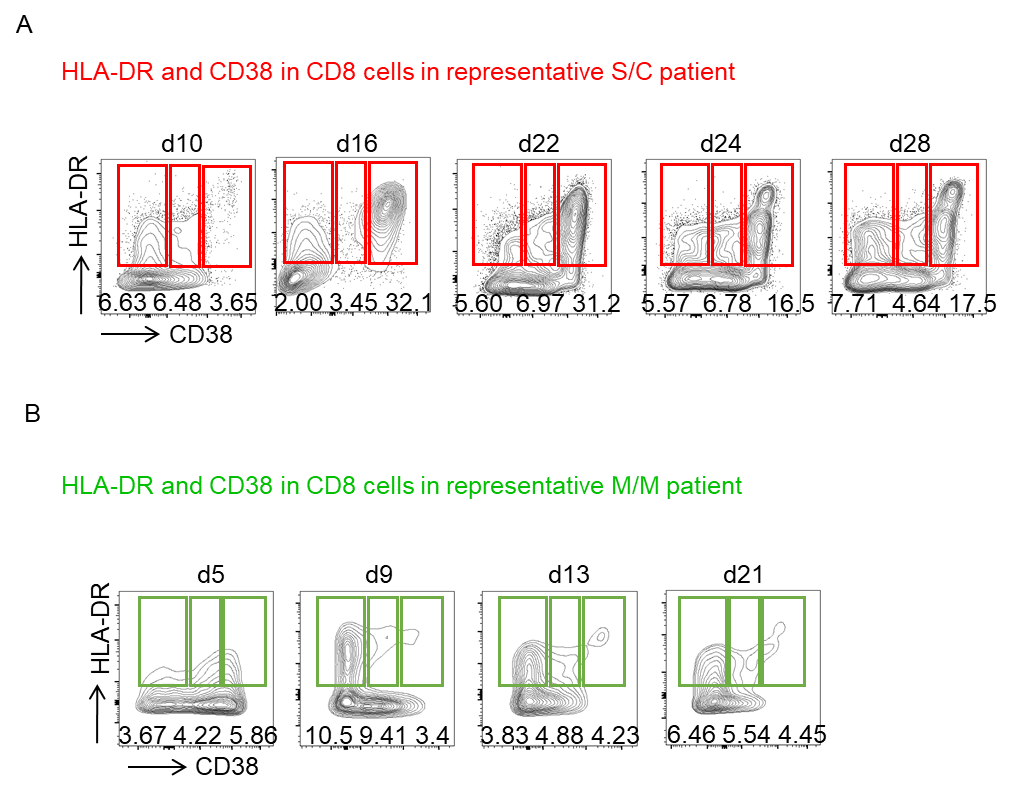
**

**Figure S1**

Representative FACS plots of HLA-DR and CD38 expression were shown at different days post onset in S/C (A) and M/M (B) patients during acute infection of COVID-19.

**Figure S2**

The ratio of HLA-DR^+^CD38^dim^ to HLA-DR^+^CD38^hi^ in CD8 was calculated by the percentage of HLA-DR^+^CD38^dim^ and HLA-DR^+^CD38^hi^ at 2-3 weeks post onset. P values were obtained by Mann–Whitney U test. ***P < .001

**
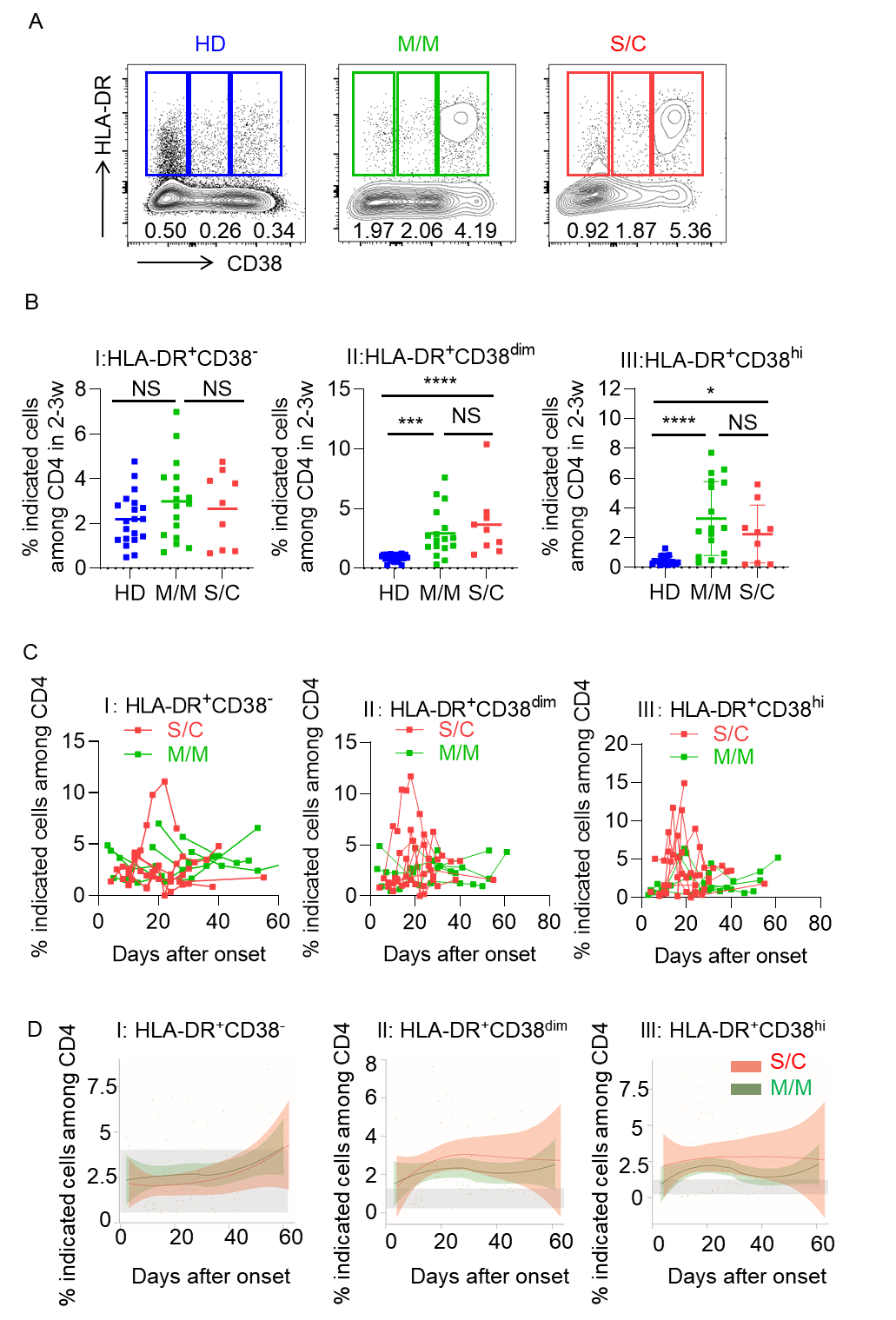
**

**Figure S3.** **HLA-DR^+^CD38^hi^CD4^+^T cells during acute infection of COVID-19.**

(A) Representative FACS contour plots showed three subpopulations of HLA-DR^+^ CD4^+^ T cells from healthy donor and COVID-19 patients: HLA-DR^+^CD38^-^ (I), HLA-DR^+^CD38^dim^ (II), HLA-DR^+^CD38^hi^ (III). (B) Scatter dot plots of three subsets of HLA-DR^+^ CD4^+^ T cells from healthy donors and patients within 2-3 weeks post onset (n = 9-20 each group). P Values were obtained by unpaired two-tailed Student’s t tests repeated measures by one-way ANOVA test followed by Tukey’s multiple comparisons test. *P < .05, ***P < .001 , ****P < .0001 (C) Longitudinal data of three CD4 subsets were graphed for 8 S/C and 7 M/M patients with three time points at least. (D) Temporal changes of three CD4 subsets in M/M (n =32) and S/C (n =10) groups during hospitalization were shown. The 95% confidence interval indicated by colored areas. The normal range of each population was gray shaded region.


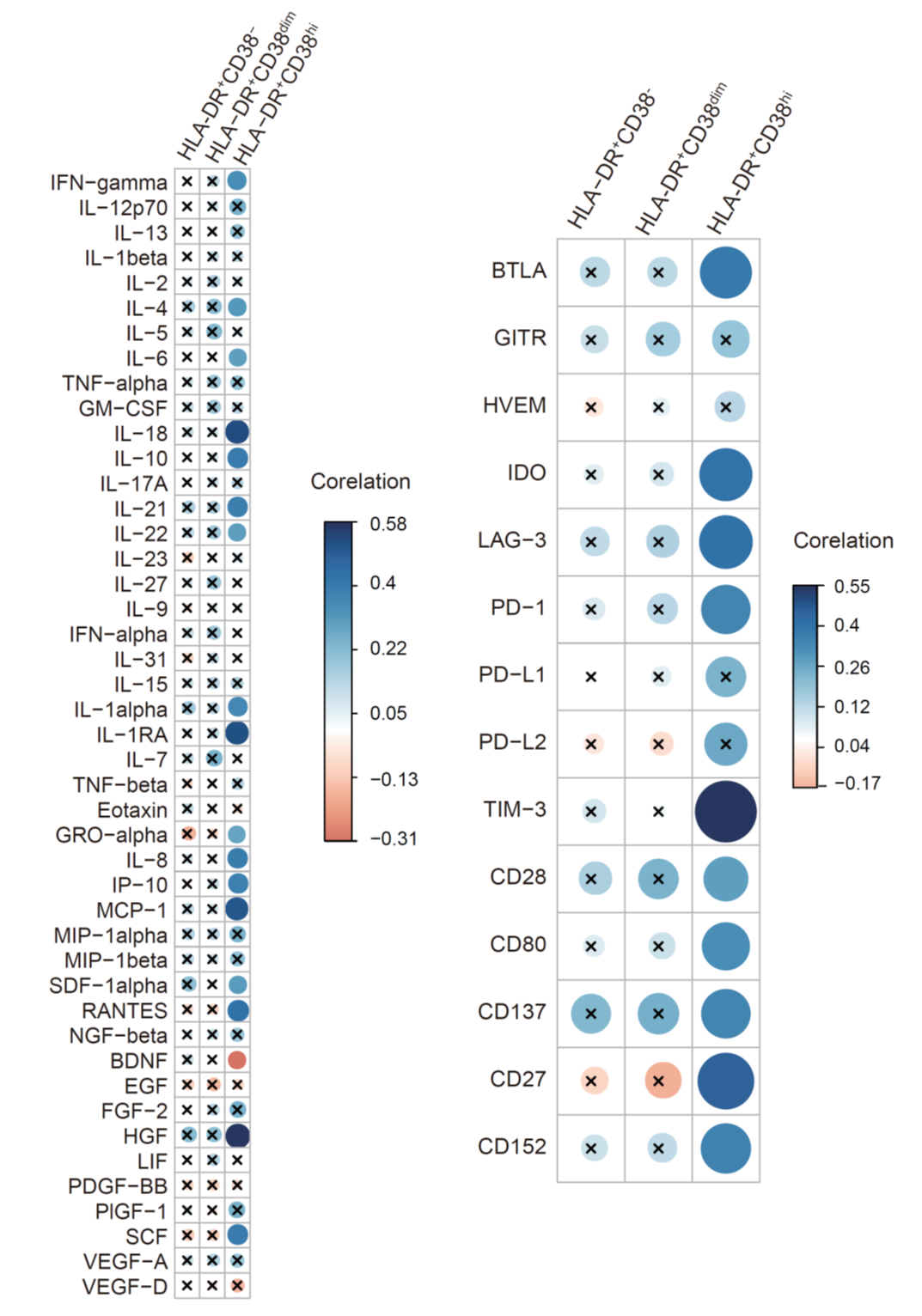


**Figure S4.**

Correlation heatmap of three CD8 subsets with 45 cytokines concentrations (left) and soluble checkpoint molecules concentrations (right) were shown. Longitudinal data were collected from 27 patients at different time points during hospitalization. P and correlation coefficient values were obtained using the Spearman’s correlation test. The circle size is proportional to the correlation coefficient value. Blue circle: positive correlation; red circle: negative correlation; values with no significant correlation are marked with a black cross.

**Table S1: Demographics and baseline** **characteristics of patients infected with SARS-CoV2.**

| Characteristics | Total  (n = 42) | Mild/Moderate patients (n = 32) | | Severe/Critical patients (n = 10) | P values |
| --- | --- | --- | --- | --- | --- |
| Age (Year) | 37 (20, 75) | | 32 (20, 75) | 54 (20, 70) | 0.001 |
| Gender |  | |  |  | 1 |
| Male (%) | 21 (50.0%) | | 16 (50.0%) | 5 (50.0) |  |
| Female (%) | 21 (50.0%) | | 16 (50.0%) | 5 (50.0%) |  |
| Admission to ICU (days) |  | | / | 23.11±6.17 |  |
| SOFA score |  | | / | 2.44±0.38 |  |
| Complications (n, %) |  | |  |  | <0.001 |
| Hypertension | 5 (11.9%) | | 1 (3.1%) | 4 (40.0%) | 0.008 |
| Cardiovascular disease | 1 (2.4%) | | 1 (3.1%) | 0 | 1 |
| Chronic Pulmonary disease | 3 (7.1%) | | 1 (3.1%) | 2 (20.0%) | 0.136 |
| Diabetes | 3 (7.1%) | | 1 (3.1%) | 2 (20.0%) | 0.136 |
| Hyperlipemia | 1 (2.4%) | | 0 | 1 (10.0%) | 0.238 |
| Chronic kidney disease | 1 (2.4%) | | 0 | 1 (10.0%) | 0.238 |
| Immune disorders | 0 | | 0 | 0 |  |
| Others | 0 | | 0 | 0 |  |
| Laboratory data |  | |  |  |  |
| WBC (×10^9^/L) | 5.42±2.03 | | 5.43±1.92 | 5.39±2.45 | 0.738 |
| Lymphocyte (×10^9^/L) | 1.45±0.59 | | 1.62±0.55 | 0.88±0.32 | <0.001 |
| Neutrophil (×10^9^/L) | 3.58±1.96 | | 3.35±1.71 | 4.31±2.59 | 0.440 |
| Monocyte (×10^9^/L) | 0.34±0.16 | | 0.39±0.15 | 0.19±0.09 | <0.001 |
| Hemoglobin (g/L) | 140.5±14.59 | | 143.1±14.16 | 132.1±13.3 | 0.035 |
| Platelets (×10^9^/L) | 213.95±82.05 | | 231.97±80.13 | 156.3±60.86 | 0.004 |
| CRP (mg/L) | 20.27±38.06 | | 5.66±11.09 | 70.58±54.12 | 0.004 |
| LDH (U/L) | 231.52±121.12 | | 197.66±61.84 | 441.44±189.0 | <0.001 |
| PT (s) | 12.37±0.82 | | 12.24±0.78 | 12.79±0.84 | 0.065 |
| APTT (s) | 31.73±3.65 | | 32.21±3.15 | 30.25±4.79 | 0.251 |
| D-dimer (mg/L) | 0.29±0.384 | | 0.18±0.08 | 0.63±0.68 | 0.017 |
| Blood Potassium (mmol/L) | 3.52±0.31 | | 3.56±0.31 | 3.37±0.28 | 0.084 |
| Blood sodium (mmol/L) | 138.31±3.04 | | 139.45±2.07 | 134.67±2.86 | <0.001 |
| Serum creatinine (μmol/L) | 69.7±17.73 | | 70.53±17.17 | 67.03±20.14 | 0.512 |
| Albumin (g/L) | 44.16±4.86 | | 46.21±3.09 | 37.61±3.49 | <0.001 |
| ALT (U/L) | 28.89±24.23 | | 25.12±23.40 | 40.98±23.97 | 0.004 |
| AST (U/L) | 31.27±26.42 | | 24.78±20.32 | 52.04±33.58 | <0.001 |
| SAA (mg/L) | 79.45±124.87 | | 24.43±51.75 | 262.86±123.13 | <0.001 |

Note: WBC: white blood cells; CRP: C-reactive protein; LDH: lactate dehydrogenase; PT: prothrombin time; APTT: activated partial thromboplastin time; ALT: alanine aminotransferase; AST: Aspertate Aminotransferase; SAA: serum amyloid A; SOFA: sequential organ failure assessment.
